# Burdens and psychosocial consequences of the COVID-19 pandemic for Austrian children and adolescents

**DOI:** 10.3389/fpsyg.2022.971241

**Published:** 2022-11-11

**Authors:** Esther-Sevil Eigl, Sebastian Stefan Widauer, Manuel Schabus

**Affiliations:** Department of Psychology, University of Salzburg, Salzburg, Austria

**Keywords:** COVID-19 pandemic, coronavirus, collateral damage, mental health, strain, fear, pandemic, SARS-CoV-2

## Abstract

**Objectives:**

The negative psychosocial effects of the COVID-19 pandemic are becoming increasingly apparent. Children and adolescents in particular, were affected and torn away from their daily life routines. The aim of our survey is to evaluate the psychosocial burden and impairments of children and adolescents in Austria during the COVID-19 pandemic by using cross-sectional analysis.

**Setting:**

An Austrian-wide online survey was conducted from 21 February to 19 April 2021 for children and adolescents. The questionnaire was distributed widely using the national press agency and public media.

**Participants:**

Using an online questionnaire, 5,483 children and adolescents between 6 and 18 years of age were sampled.

**Outcome measure:**

Quantitative responses to questions regarding the children’s feelings, worries, and needs concerning the COVID-19 pandemic were measured. Furthermore, the children were sampled for subjective risk perception as well as their sleep quality.

**Results:**

Most children reported a high degree of fear due to the pandemic, especially female (48.1%) participants being under more emotional strain than their male (35.9%) counterparts. Associated with this, we found a strong overestimation of COVID-19-associated hospitalization likelihood (>100-fold) across all age groups. In addition, an alarming lack of positive perspective during the ongoing pandemic is evident across all age groups, including the youngest participants aged 6–10 years. Feelings of anger and annoyance (58.2%), loneliness (46%), and sadness (42.7%) are reported much more frequently than before the pandemic. On the other hand, only 15.6% reported feeling well (or even better; 2%) since the COVID-19 pandemic. Last but not least, our study shows an alarming 37% of children and adolescents who now report poorer sleep quality than before the pandemic.

**Conclusion:**

The results of this survey indicate the high burden and emotional strain for children and adolescents during the pandemic. Personal contact with friends and family is mentioned as the most protective factor for their mental health. The study results underscore the need for immediate action to limit the collateral damage that has already occurred on a psychosocial and developmental level among younger generations worldwide.

## Highlights

- Literature indicates that specifically children and adolescents suffer from the COVID-19 pandemic and associated restrictions, such as school closures, fewer social contacts, and/or less physical exercise.
- Having no real lobby, the needs and concerns of children and adolescents have unfortunately been widely neglected in the political and public discourse.
- This study gives voice to this vulnerable group and emphasizes the alarming degree to which Austrian children and adolescents are under psychological strain since the start of the pandemic.

## Introduction

The COVID-19 pandemic has been dominating the world and the lives of many, with lockdowns starting in Europe around February/March 2020. There has been much discussion about the consequences for health that are associated with COVID-19 directly and indirectly. Unfortunately, an important aspect of health has been largely neglected worldwide, namely, the mental health aspect in the general population (Gloster et al., 2020; AlGharaibeh and Gibson, 2021; Panda et al., 2021) and especially in children and adolescents (Liang et al., 2020; Zhou et al., 2020; Ravens-Sieberer et al., 2021b). From our perspective, it is worrisome that the interplay between mental well-being and somatic health has been discussed and addressed so little during the current pandemic. According to the WHO (World Health Organization [WHO], 2018), “Mental health is an integral component of health; in fact, there is no health without mental health.” Therefore, this survey aims to take a closer look at the psychosocial impact of the COVID-19 crisis on Austrian children and adolescents. More specifically, we address their subjective burdens, feelings, and the nature of their fears, but also what helped them best through the pandemic or how they subjectively perceived the risk of getting severely ill due to the coronavirus (SARS-CoV-2) infection.

### Psychosocial consequences of the pandemic

As early as November 2020, an international multicenter study documented that COVID-19 home confinement negatively affects mental well-being (Ammar et al., 2020). In fact, 12.89% more individuals reported low psychological well-being during home confinement as compared to before. In addition, the results of the mood and feelings questionnaire used showed a significant increase of burden by 44.9%, and also more individuals (+10%) were reporting depressive symptoms during home-confinement as compared to before.

In this regard, it is also worth mentioning that data from over 500,000 coronavirus hospitalizations in the USA indicate that mental factors, such as anxiety, are among the primary driving forces determining the course of a SARS-CoV-2 infection. Indeed, among COVID-19 hospitalized patients, the strongest risk factors for death were obesity, anxiety, and fear-related disorders (Kompaniyets et al., 2021).

Especially overlooked are the psychosocial consequences that children and adolescents suffer from in reaction to COVID-19 countermeasures, including school closures (Liang et al., 2020; Ravens-Sieberer et al., 2021a; Zhou et al., 2021). Social distancing and isolation lead to fewer social contacts which is especially difficult to handle in times of elevated stress and crisis. The negative effects that come with this social deprivation often affect children and adolescents the strongest. Whereas many adults kept working and may maintain social contacts to some degree, the effect of school closures, or the prohibition to visit friends or work out in sports clubs may have hit this vulnerable group the strongest. It is important to emphasize that children and adolescents are at a sensible phase in their early development, where the social environment may be vital for (later) well-being, including brain development, construction of self-concept, and mental health (Orben et al., 2020). In particular, the “social brain” is shaped until late adolescence (Blakemore, 2012) and learns how to draw inferences about other people, their intentions, feelings, and thoughts.

Thus, the negative psychosocial impact of the coronavirus pandemic was certainly not limited to people who directly suffered from a severe (somatic) course of the disease. Negative psychosocial effects, such as posttraumatic stress symptoms, confusion, and anger, as a consequence of quarantine measures, were noted on a population level throughout the world when people were cut from daily life routines due to governmental restrictions to contain the pandemic (Brooks et al., 2020). From a psychological point of view, one can even argue that the COVID-19 pandemic triggered a second pandemic, namely, one of fear, anxiety, and depression (Yao et al., 2020); and today, there is plenty of scientific evidence documenting the vast psychosocial effects associated with the pandemic and its governmental countermeasures (Brooks et al., 2020; Dubey et al., 2020; Sun et al., 2020).

### COVID-19 and psychosocial outcomes in Austria and Europe

As far as Austria is concerned, an early study conducted during the first wave in April 2020 compared psychological symptoms during the lockdown to pre-COVID-19 in Austria and found that the prevalence of depressive symptoms increased about fivefold (Pieh et al., 2020). Likewise, anxiety symptoms increased threefold compared to before the COVID-19 pandemic. Overall, life satisfaction and well-being decreased compared to pre-COVID-19. Especially worrisome is that, for each aspect of mental health tested, the younger adult groups (<35 years) had the worst test scores. In a related study, Italian and Spanish parents were asked about the well-being of their 3–18-year-old children (Orgilés et al., 2020). Specifically, information was obtained on how quarantine affected their children and themselves compared to the period before home confinement. It was found that 85.7% of parents reported observing changes in their children’s emotional state and behavior. The most common symptoms included difficulty concentrating (76.6%), boredom (52%), irritability (39%), restlessness (38.8%), nervousness (38%), and loneliness (31.3%). In addition, children spent more time on their cell phones, did fewer sports, and slept more during the day.

A more representative study of 7–17-year-old children and adolescents in Germany also reported significant negative psychological effects (Ravens-Sieberer et al., 2021a). Data obtained from this survey were compared with data from the representative BELLA cohort study performed before COVID-19 (Ravens-Sieberer et al., 2015; Otto et al., 2020). Alarmingly, two-thirds of the children and adolescents surveyed reported suffering severely over the course of the pandemic. Respondents had significantly lower health-related quality of life (HRQoL) scores (40.2 vs. 15.3%), higher anxiety scores (24.1 vs. 14.9%), and more mental health problems (17.8 vs. 9.8%) compared to before the pandemic (Ravens-Sieberer et al., 2021a). Especially disadvantaged children with migration backgrounds, low socio-economic status, and restricted housing were substantially more affected.

One possible reason why the needs of children and adolescents have been overlooked for so long may be that there was a strong focus on the negative somatic health consequences due to COVID-19, although children were known to be much less affected. Axfors and Ioannidis (2022) reviewed 13 seroprevalence studies conducted in 11 countries to identify the mortality rate of infections and found that in the 0–19 age cohort, the infection mortality rate is only about 0.0009% and increases steeply with age in community-dwelling elderly people (70+) to 2.2% or for the elderly overall (including nursing homes) to 4.0% (accounting for 5% monthly seroreversion). It is important to note that the infection fatality rate (IFR) differs from the often publicly reported case-fatality rate (CFR), because the IFR indeed considers the whole population and thereby also takes into account people with a mild or asymptomatic disease course that never saw a doctor or hospital because of their SARS-CoV-2 infection. It seems that the burden carried by children and adolescents was therefore longer “under the radar” of scientists and especially politicians and that there is a problematic bias in the sense that somatic health is seen as widely independent of mental health. Another reason could be that children and adolescents do not have an active lobby to significantly influence policymakers regarding their particular concerns and needs.

### COVID-19 counter-measures and their effects on well-being

It is well established that lockdowns and quarantine measures are major risk factors for mental health (Brooks et al., 2020; Xie et al., 2020; Zhou et al., 2021) and especially for children and adolescents (Fegert et al., 2020). In the acute pandemic phase, the main burdens on them were social distancing, increased pressure on their families, and associated domestic violence and physical abuse, as well as limited access, to support structures, such as sports clubs, schools, or other places to meet their peers (Green, 2020; Sacco et al., 2020; Sidpra et al., 2020; Loiseau et al., 2021). After the pandemic, we will need to deal with the long-term consequences related to chronic stress, anxiety, and another increase in domestic violence related to economic challenges following the pandemic. Disadvantaged and marginalized children and adolescents will be disproportionately affected by these COVID-19-related mental health risks, as already documented (Ravens-Sieberer et al., 2021a).

Various measures have been implemented to contain the COVID-19 crisis. School closures in particular have disrupted the lives of children and young people. The German Youth Institute conducted a mixed-method survey on the situation of children and young people in the spring/summer of 2020 (Langmeyer et al., 2020). Among other things, the survey dealt with homeschooling and found that half of the parents interviewed reported significant burdens due to the organization of homeschooling in their families. Success was highly dependent on how well organized the schools were; only if schools were able to provide a good structure for homeschooling, with good materials, a concept, and perspective, was the situation perceived as acceptable.

In Austria, COVID-19 counter-measures have now been in place for more than 2 years (since 16 March 2020). These include, in addition to the general hygiene guidelines and distance recommendations, repeated lockdowns, school closures, closures of restaurants, sports, and recreational facilities, curfews, contact bans, and a vaccination mandate with significant fines for non-compliance that was in place by 1 February 2022. To add the time dimension of our study, note that 2 weeks before the start of data collection, many of the measures that had been in place (such as the closure of shops and schools) were lifted (i.e., on 8 February 2021) in Austria. In total, the three lockdowns up to this point lasted 165 days in Austria. During all of this time, social contacts were massively restricted and there were strict and mandatory curfews for all citizens. An earlier survey of our group already focused on the knowledge and attitudes of the Austrian (Swiss and German) population toward the COVID-19 pandemic in people aged 18 years and older and indicated significant worry in the adult general population (Schabus et al., 2022). In order to focus on possible psychosocial effects in children and adolescents, we started the online survey “Now it’s your turn to speak up!” on 21 February 2021 for Austria. The goal of this study was to document the psychosocial burden and give children aged 18 and younger a voice and make their view on the pandemic more visible to the scientific community, to authorities, and beyond.

## Materials and method

A total of 5,483 children and adolescents between the ages of 6 and 18 participated in the Austria-wide “Now it’s your turn to speak up!” (In German, “Jetzt Sprichst Du!”) survey between 21 February and 19 April 2021. All participants gave informed consent before filling out the questionnaire. The survey was made public *via* the Austrian Press Agency (APA), public television (ORF), print media, and various youth organizations and schools.

The survey was a self-designed *ad hoc* questionnaire with the main aim to capture the burdens and psychosocial consequences of the COVID-19 pandemic for Austrian children and adolescents. In addition, we were interested in how this group perceived that special time with school closures, lockdowns, and often high amounts of fear in their immediate social environment. Finally, we were interested in comparing subjectively estimated risks for coronavirus (SARS-CoV-2)-related hospitalization with actual reference data for such hospitalization at that young age and evaluate how far individuals from more (coronavirus-related) anxious social environments differed in their subjectively perceived fears, risk assessment, and social habits. More specifically, the survey consisted of 36 questions and was available *via* the online survey tool “LimeSurvey” (version 3.26). The first five questions gathered demographic information about the participants (i.e., sex, age, state, school, and relationship status). After that, nine questions assessed school-related experiences during the coronavirus pandemic (e.g., “How much do you miss everyday school life?”; “How much do you like home-schooling in comparison to ‘normal’ school life?”). Furthermore, there were nine questions on the burden, fears, and resources in connection to the pandemic (e.g., “How do you feel since the ‘coronavirus’ outbreak?”; “What helps you the most in the current situation?”), two questions about social contacts during the pandemic (e.g., “How often did you see your relatives since the beginning of the coronavirus pandemic in comparison to before?”), and 11 further questions about personal risk perception, source of information regarding the COVID-19 pandemic, attitudes of relatives, social environment, and contact with the disease (e.g., “Where do you mainly get your information about the coronavirus pandemic from?”; “What do your parents think about ‘Corona’?”; “Has anyone of your family or friends had Corona since the outbreak of the pandemic?”). Optionally, the participants could also complete an additional second part with 25 questions focusing on changes in sleep habits and sleep quality since the pandemic [for more see Bothe et al. (under review)^1^]. For more details, see the original questionnaire available at https://doi.org/10.17605/OSF.IO/T5RXB.

For data-cleaning purposes, a plausibility check for data integrity was performed in addition to an age check before the data were analyzed. Specifically, answers to the question “What do you think: Out of 1,000 students who are like you, how many of them will get seriously ill with the coronavirus and end up in hospital in the next 12 months?” were compared to the same persons’ answers to the associated question “How great do you think the risk is for you personally of having to go to hospital because of the coronavirus?” (4 levels from “very great” to “not at all”). Persons who estimated that max. 1 case (per 1,000) would have to be hospitalized, were only included if they also rated the subjectively assessed risk of having to be hospitalized because of the coronavirus as “minimal” or “not at all.” Furthermore, persons who stated that at least 412 of 1,000 (95th percentile) children would have to be hospitalized were only included in the final analysis if they also assessed their subjective risk of a coronavirus-related hospitalization as “very great” or “great.”

The excluded data were reviewed and uncovered that 291 subjects were excluded because of invalid age (<6 or ≥19), and another 188 subjects because they did not pass the data integrity check outlined above; some of the participants were excluded for both reasons.

The final sample consisted of 5,008 children and adolescents between 6 and 18 years of age who answered the survey up to the last question of the general part. The second part of the survey on sleep habits was completed by 2,290 children and adolescents and is matter of a separate publication in preparation (see text footnote 1). When reporting questions with ranks as answer options, the frequencies of the first three (top ranked) categories were added up for the analysis. If a category was already selected as rank 1, indicating that no other options were applicable (e.g., “I’m not really afraid”), only the first rank was reported. In addition to the questions that had to be ranked by priority, we also had 20 Likert-scale variables where we for example asked, “Does the current situation with the coronavirus scare you?” on a Likert scale from “1 = very much” to “4 = not at all.”

## Results

Of the 5,008 children and adolescents, 60.9% were female, 37.9% male, and 1.2% diverse. The sample was divided into three age groups: 6–10-year-old “elementary school students” (*n* = 949), 11–14-year-old “middle school students” (*n* = 1,930), and 15–18-year-old adolescents (*n* = 2,129). When asked (from 1 = very much to 4 = not at all) if the children and adolescents were “scared by the current situation concerning the coronavirus,” 48.1% of the girls and 35.9% of the boys answered that the situation scared them (“very much” or “somewhat”). Here, girls were significantly more afraid because of the current situation than boys (*Z* = −8.890, *p* < 0.001), which is driven by the age groups above age 10, as the youngest age group (6–10 years) did not differ in their anxiety levels depending on sex (*Z* = −0.828, *p* = 0.407).

**FIGURE 1:**
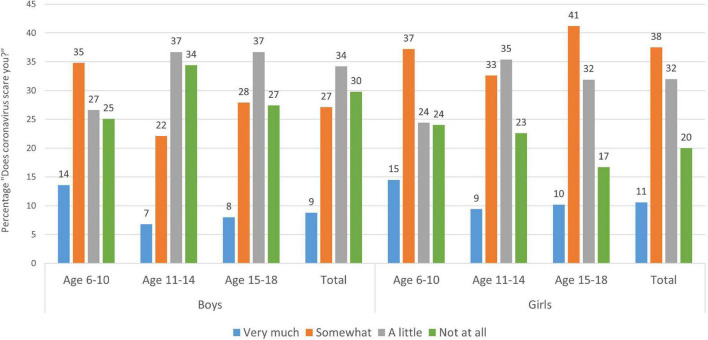
Distribution of children’s and adolescents’ answers to the question “Does the current situation regarding the coronavirus scare you?” by age group and gender. Boys to the left, girls to the right with each age group (6–10 years, 11–14 years, and 15–18 years), and the total grand mean to the right. Each age category sums up to 100% (rounded values). Figures are modified and reproduced (CC BY 4.0) from Schabus and Eigl (2021).

Comparing age groups, it is noticeable that elementary school children show significantly greater fear with regard to the coronavirus than the 11–14-year-old children (2.83 ± 0.928; *Z* = −5.903, *p* < 0.001). Every second elementary school child feels frightened by the current situation (“very much” or “somewhat”) (for detail see Figure 1). Statistically speaking, 15-18-year-old adolescents are comparable to the youngest ones in terms of their level of concern regarding the pandemic (*Z* = 0.897, *p* = 0.370).

**FIGURE 2:**
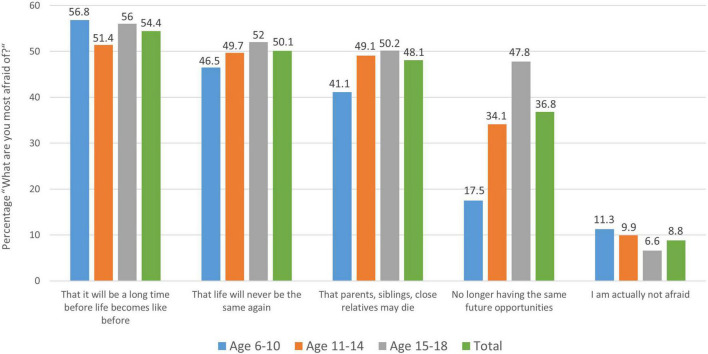
Distribution of children’s and young people’s answers to the question “What are you most afraid of in relation to the coronavirus?” by age group. Here the mean of each age group as well as the total grand mean are shown next to each other for the most prevalent answer options chosen by the children and adolescents. Note that only about 8.8% of the participants state that they are “actually not afraid” and therefore chose none of the fear-related answer options.

When asked about the nature of these fears, the following ones are primarily mentioned: “That it will be a long time before life will be like it was before” (54.4%), “that life will not be like it was before at all” (50.1%), “that parents, siblings or close relatives might die” (48.1%), and that they will “no longer have the same future opportunities or job opportunities” as they had “before the coronavirus” (36.8%); only 8.8% of children and adolescents said they actually had none of these fears. The fear of no longer having the same future opportunities is particularly pronounced among adolescents aged 15–18 (47.8%; see Figure 2).

**FIGURE 3:**
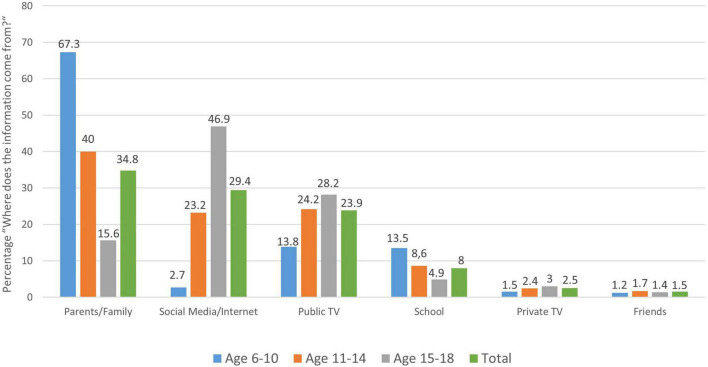
Distribution of children’s and adolescents’ responses to the question: “Where do you mainly get your information about the coronavirus from”, by age group.

Next, we asked ourselves where the children and adolescents mainly get their information about the coronavirus from. It is interesting that elementary school students primarily obtain their information regarding the coronavirus from their parents and family (67.3%), whereas adolescents primarily use “social media” or the internet as a source of information (46.9%); public television and school play a comparatively minor role in that young age group (see Figure 3).

**FIGURE 4:**
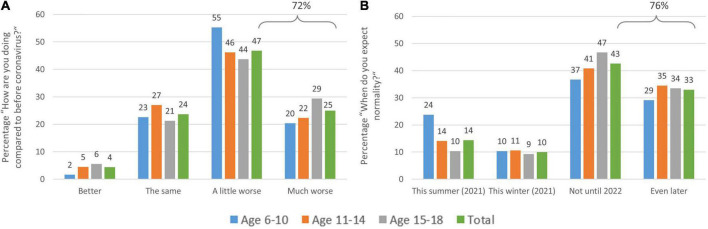
Distribution of children’s and adolescents’ responses to the question: **(A)** “How are you doing compared to before coronavirus?” and **(B)** “When do you think life will be relatively ‘normal’ again?” by age group. **(A)** About 72% of the sample are doing worse and **(B)** expect normalization of the situation only in the far future (surveyed in February–April 2021).

When children and adolescents are asked how they are doing compared to before the coronavirus pandemic (from 1 = better to 4 = a lot worse), 71.8% say they are doing “a lot” (25%) or “a little” (46.8%) worse than before the coronavirus (see Figure 4A). Girls (*M* = 2.95 ± 0.813) do significantly worse than boys (*M* = 2.87 ± 0.800) in this regard (*Z* = −3.76, *p* < 0.001), which is driven by the older two age groups (above age 10), as in the youngest age group there is no difference between boys and girls regarding their emotional state since the coronavirus outbreak (*Z* = −0.774, *p* = 0.439). Sex differences are revealed in the older age groups 11–14 years (*M*_*F*_ = 2.91, *SD*_*F*_ = 0.82, *M*_*M*_ = 2.81, *SD*_*M*_ = 0.78; *Z* = −2.555, *p* = 0.011) and 15–18 years (*M*_*F*_ = 2.99, *SD*_*F*_ = 0.85, *M*_*M*_ = 2.90, *SD*_*M*_ = 0.87; *Z* = −2.061, *p* = 0.039).

When asked (in February–April 2021) when they think “life will be relatively ‘normal’ again,” (from 1 = this summer (2021) to 4 = later than 2022), 75.6% say they do not expect a return to normality until 2022 or later (cf. Figure 4B) with 33% even expecting 2023 or later. This can be taken as an expression of a certain lack of perspective among children and young people and likewise across all age groups at that point of time. This is particularly strong in girls (*M* = 3.01 ± 0.97) in comparison to boys (*M* = 2.83 ± 1.04; *Z* = −5.83, *p* < 0.001), while here as well, this difference is driven by the older age groups (11–14 years, *M* = 2.96 ± 1.007, *Z* = −4.05, *p* < 0.001; 15–18 years, *M* = 3.03 ± 0.92, *Z* = −3.99, *p* < 0.001). In the youngest age group, there is no difference between girls (*M* = 2.71 ± 1.13) and boys (*M* = 2.71 ± 1.12; *Z* = −0.10, *p* = 0. 921).

**FIGURE 5:**
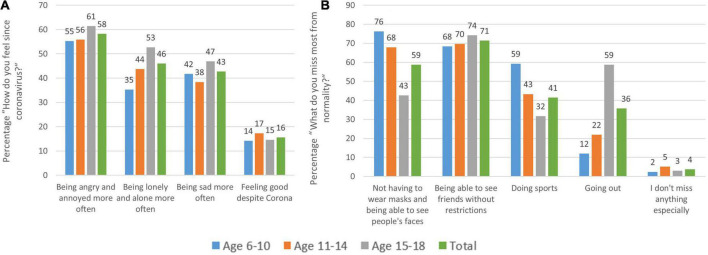
Distribution of children’s and adolescents’ answers to the question **(A)** “How do you feel since the coronavirus?” and **(B)** “What do you miss most from normality?” by age group. **(A)** Many young participants report being more angry, lonely, or sad than before the coronavirus. What they miss most **(B)** is not wearing masks, meeting friends, and doing sports as well as going out (in adolescents) with only 4% not missing anything.

Furthermore, the survey asked about the prevailing feelings since the coronavirus pandemic. The most frequently mentioned feeling since the COVID-19 pandemic was “being angry and annoyed more often” (58.2%), followed by “being lonely and alone more often” (46%) and “being sad more often (42.7%).” 15.6% feel “good despite the coronavirus” (13.6%) or “even better” (2%; see Figure 5A). When asked about the things that children and adolescents miss most from normality, the answer most frequently given was “being able to meet friends without restrictions” (71.4%), followed by “not having to wear masks and being able to see people’s faces” (58.7%), and “being able to do sports” (41.4%). Adolescents report “going out” as the second most important thing missing since the pandemic (58.7%) (see Figure 5B). Across all age groups, children and adolescents report that they miss the normal school day “extremely” (29.8%) or “quite a bit” (31.7%). Elementary school students, in particular, suffer from the current situation, with 72.2% stating that they miss everyday life “very much” (42.1%) or “quite a bit” (30.1%).

According to the children’s and adolescents’ assessment, the things that could help them most in the current situation are “meeting friends in person” (50.4%), “spending time with family” (49.2%), “spending time in nature” (39.3%) and playing with the mobile phone/computer games (28%). Chatting with friends online (18.4%) and meeting friends online (17.1%) seems to help a few of the participants. A total of 4.4% even state that at the moment nothing helps them to feel better.

We also examined whether it makes a difference that children and adolescents live in an environment with high coronavirus-related anxiety or one with low anxiety of the parents. For this purpose, the variable “What do your parents think about the coronavirus?” was used for categorization, and children and adolescents who felt that their parents thought the coronavirus was “very dangerous” or “dangerous” were assigned to the “anxious” group. Children who indicated that their parents considered the coronavirus to be “not at all dangerous,” “not very dangerous,” or “like the flu” were defined as the “not anxious” group. Across age groups, the “anxious group” was more likely to report having “almost not seen their relatives at all” (44.6 vs. 33.6% for the non-anxious group) and being more afraid of the virus (47.1 vs. 40.1%). When assessing the dangerousness of the SARS-CoV-2 pathogen, 84% of the anxious group said themselves that the coronavirus was “very dangerous” or “dangerous,” with only 13% of the non-anxious group agreeing with this assessment. Also, children and adolescents in the anxious group estimated the risk of COVID-associated hospitalization as much higher (15.2%) than the children from non-anxious environments (4.7%). A similar picture emerged when asking for absolute numbers, that is how many children “like you” would be estimated to need hospitalization following a SARS-CoV-2 infection (4.28 vs. 1.52% children/adolescents “who are just like you”). The greatest fears mentioned by children and adolescents also differed significantly between these two groups. Whereas the more fearful group stated that their primary fear that “parents, siblings or close relatives could die,” (61%) or “that parents, siblings or close relatives could fall ill” (42.6%), the non-anxious group is primarily concerned that “life might not be the same as before” (61.1%) or that they might “no longer have the same future.”

Furthermore, the children and adolescents were asked about their assessment of the risk posed by the coronavirus (SARS-CoV-2): “What do you think: Out of 1,000 students who are like you, how many of them will get seriously ill with the coronavirus and end up in the hospital in the next 12 months?” Interestingly, the risk of being hospitalized for SARS-CoV-2 infection is massively overestimated by children (1.2% 6–10 years, 3.3% 11–14 years; 5% trimmed means) and adolescents (3.1%), even though the estimated risk is less than 1 in 10,000 (<0.01%) among children and adolescents with health risk factors or less than 1 in 40,000 (<0.004%) among those below 19 years of age without health risk factors (Clift et al., 2020; Berner et al., 2021).

We also asked children how high they perceive their personal risk for the need for hospitalization due to COVID-19 (from 1 = very high to 4 = not at all). Again here, girls are more concerned (*M* = 3.25 ± 0.695) than boys (*M* = 3.32 ± 0.719; *Z* = −4.528, *p* < 0.001) with the overall pattern showing “minimal” concern of being personally affected by COVID-19. While in the youngest age group, there is no difference between girls (*M* = 3.41 ± 0.706) and boys (*M* = 3.41 ± 0.756; *Z* = –0.559, *p* = 0.576), in the older age groups girls are slightly more concerned than boys. (11–14 years; *Z* = –2.655, *p* = 0.008; 15–18 years; *Z* = −2.834, *p* = 0.005).

As another reference, the University of Oxford (UK) QCovid^®^ algorithm (Clift et al., 2020), from which the following data is derived, is an evidence-based model that incorporates factors such as age, sex, ethnicity, or pre-existing conditions to estimate the risk of COVID-19-associated hospitalization or death. At https://qcovid.org, this risk can be calculated for any individual aged 19 and above. An illustrative example can be a 19-year-old man (180 cm, 80 kg) with no pre-existing condition (0.0023%) or with type 1 diabetes and asthma with a 0.0093% risk of hospitalization or a 1:200.000 risk for COVID-19-associated mortality.

The fear that a parent or close relative will die—which is one of the primary fears in children and adolescents (Figure 2)—also appears to be overestimated by children and adolescents with the actual risk of hospitalization being about 0.21% (1 in 469) and that of COVID-19-associated death is about 1 in 1,344 (0.07%) for a typical grandparent with a pre-existing condition (model calculated with a 75-year-old woman with type 2 diabetes, asthma, 165 cm, 70 kg (Clift et al., 2020)).

Last but not least in the second part of the questionnaire (*n* = 2,290; for more details (see text footnote 1), it became evident that physical activity since the coronavirus was “very much less” or “less” (74.8% of children and adolescents) than pre-pandemic. In addition, the amount of time that the children/adolescents spent in natural daylight also became significantly shorter in 44.2% of the participants. Yet, children, as well as adolescents, spent much more time with smartphones, tablets, or PCs (83.0%). Given these circumstances, also sleep suffered. More than one in three children (37%) reported poorer sleep quality since the pandemic and 38.9% of children even reported now having problems with sleep, which is quite unusual at this young age. Of those who reported sleep problems, 42.5% are plagued by difficulty falling asleep, and 20.3% by difficulty staying asleep. In addition, 9 out of 10 children/adolescents (94.3%) now go to bed later during the week. Age-wise, most coronavirus-related sleep problems are seen in adolescents (45.3%), whereas 16.4% of the youngest (6–10 years) now seem to experience more nightmares (16.3%) than before.

## Discussion

In summary, an alarming picture of psychosocial health and well-being among children and adolescents emerges. We focused on various topics and found that from all participants sampled only about 9% said that they have no fears related to the pandemic. Surprisingly, the prevailing fears were more that it will be a long way back to normality or that the future will not provide the same opportunities as before the pandemic, as compared to the fear that a close relative dies due to the coronavirus (SARS-CoV-2).

More worrisome than the subjective fear are the ratings concerning subjective well-being with more than two-thirds saying that they do worse than before. Their prevailing emotions are such of being more angry/annoyed, lonely, or sad with only 16% saying they feel good despite the pandemic. What helps them most are activities that were forbidden over extended times during the pandemic, such as meeting friends personally or doing sports. Finally, we see that besides adults (Schabus et al., 2022), also children and adolescents seem to substantially overestimate their subjective risk to get hospitalized due to a SARS-CoV-2 infection, which again contributes to their generally high anxiety levels and worsened sleep.

More specifically, we found that 48% of the girls and 36% of the boys were somewhat scared by the coronavirus situation, with girls being more afraid than boys (if aged 10 and older). In addition, it is the youngest age group (6–10) and the oldest (15–18), which show statistically more fear due to the Corona situation (in February–March 2021) than the 11–14 aged ones. We can only speculate about the cause of this, but we believe that very different reasons drive their fear. While the youngest group seems to be most worried that it will take a long time until life will be as it was before, the oldest group (15–18) states that they are worried about their future (job) opportunities given the pandemic and its aftermath.

What also becomes evident is that children/adolescents raised in families where the parents have many Corona-related fears themselves show very different results from those with low Corona-related fears.

When asking about the dangerousness of the SARS-CoV-2 pathogen for these two groups raised in a different parental environment, 84% of the “anxious group”, for example, say themselves that the coronavirus is (very) dangerous as compared to only 13% of the “non-anxious group.” This difference is also reflected in the group estimations of their personal risk for COVID-19-associated hospitalization: 15% in the “anxious group” believe that this risk is very high, while only about 5% of the “non-anxious” group do so, which in both cases is still a vast over-estimation of their true personal risk of below 1:10.000 (<0.01%) (Clift et al., 2020; Berner et al., 2021).

When asked about the nature of these fears, every second child mentions its concern that “it will be a long time before life will be like it was before” (54%), or even “that life will not be like it was before at all” (50%). Another 48% of the children/adolescents are worried “that parents, siblings or close relatives might die” due to COVID-19. These are alarming numbers, especially considering that only 8.8% of our over 5,000 surveyed children and adolescents said that they actually have none of such fears at all.

When asked more generally about their subjective well-being 25% said they are doing “a lot” and 47% “a little” worse than before COVID-19. Again, it is the girls here (aged older than 10) that are doing worse than their male counterparts.

Irrespective of sex, their prevailing feelings since COVID-19 are thereby “being angry and annoyed more often” (58% of all children/adolescents), followed by “being lonely and alone more often” (46%) or “being sad more often (43%).” Only 16% feel “good despite COVID-19” (14%) or “even better” (2%). Although, the reader might not consider this surprising when reading this in 2022 and later, we believe it is indeed an expression of loss of perspective and faith in the future, as back in February 2021 more than three-fourths of the children and adolescents in our survey said they do not expect a return to normality before 2022 or even much later.

Remarkably, more than one in three children in our survey reported poorer sleep quality than before the pandemic. Interestingly, this is an effect extending to the general population, where we saw similar results despite more reported sleep during lockdowns (Florea et al., 2021) and school closures (Albrecht et al., 2022). We believe that this indicates that lockdowns, on the one hand, helped to adapt more to one’s personal sleep schedule and sleep needs, and on the other hand documents how daytime worries transfer into sleep and lead to sleep disruptions, worsened sleep quality and ultimately less restorative sleep.

When asking what children and adolescents missed most during the pandemic, they pointed at things that were often restricted during those times: 71% missed “being able to meet friends without restrictions” the most, followed by “not having to wear masks and being able to see people’s faces” (59%), or “being able to do sports” (41%). Adolescents also mentioned “going out” as the second most important thing missing since the pandemic (59%). The latter should be taken as seriously as the previous concerns, as for adolescents this is more than a simple leisure activity. At that age, it is actually a part of their social lives and an important cornerstone in their development and for becoming independent individuals. To our surprise, we found that - across all age groups - children and adolescents missed the normal school day “extremely” (29.8%) or “quite a bit” (31.7%) with elementary school children suffering by far the most; 72% stated that they miss everyday life “extremely” (42%) or “quite a bit” (30%).

Together with the documented devastating impact of lockdown and school closures on children’s and adolescents’ health and well-being (Rajmil et al., 2021; Abrams et al., 2022; Viner et al., 2022), this calls for a re-evaluation of containment policies, including school closures such as in the past. Rajmil et al. (2021) reviewed 22 studies from Australia, Spain, and China and found a school closure-related increase in depressive symptoms and a decrease in general life satisfaction. They also report a decrease in physical activity and an increase in unhealthy food consumption during these times. Also, the literature review highlights other worrisome collateral damages such as an increase in child mortality or a steep decrease in immunizations (besides SARS-CoV-2) in low-income countries. What is even more alarming, is the documented decrease in child protection referrals and at the same time a dramatic increase in abuse-related injuries and other injuries indicative of child abuse during the pandemic (Rajmil et al., 2021; Abrams et al., 2022; Viner et al., 2022).

Another report with over 79,000 children and adolescents comes to quite similar conclusions and states that between 18 and 60% of children/adolescents scored above risk thresholds for distress, particularly anxiety and depressive symptoms (Viner et al., 2022). It is also increasingly documented that school closures had a disproportionately negative impact on those who are disadvantaged anyway and who already face adverse social determinants, such as poverty, race, or ethnicity (Abrams et al., 2022). In summary, available studies have shown that lockdowns and school closures have dramatic effects on child health and well-being in the short but most likely also in the long term.

From a psychological point of view, one top priority must thus be to strive for rapid normalization, especially for children and adolescents, including leisure and sports activities, open schools, and social contact. The psychosocial aftereffects of the pandemic are difficult to assess today, but it is clear that the atmosphere in which children and adolescents grew up in the past 2 years is highly challenging and will likely result in developmental problems such as a surge of anxiety disorders, attachment problems, and other mental health issues besides psychosomatic complaints and consequences, such as truancy. We do not want to speak of a “lost generation,” but the burden which is seen in current data concerning psychosocial aspects of health in this young generation is at least alarming (Dubey et al., 2020; Liang et al., 2020; Orgilés et al., 2020; Otto et al., 2020; Pieh et al., 2020, 2021).

Turning to the protective factors, our present study indicates that “meeting friends in person” is subjectively the most helpful factor for children and adolescents across all age groups (50% rank it consistently in their top three priorities) with online chats and online meetings obviously not being able to compensate for personal social contacts (only 17–18% rank it as helpful).

Given the above-reviewed effects, it would also be advisable to expand psychological as well as counseling offers at all levels for children and adolescents. An approach worth mentioning is for example psychotherapy to improve the well-being of disadvantaged adolescents. In a study by Shahrokhian et al. (2022), adolescents between the age of 15 and 18 were offered online cognitive behavioral therapy (CBT; 10 sessions, each 120 min), which led to greater well-being and less stress compared to a control group. This study is just one example that shows that psychological and psychotherapeutic support, in general, must be supported and considered more seriously to help people cope with mental challenges associated with the pandemic. Another aspect we are concerned about is that the worst in the psychosocial domain may be yet to come, as the economic consequences in the aftermath of the pandemic will not only add pressure on families and will indirectly contribute to increases in domestic violence (Sacco et al., 2020; Sidpra et al., 2020; Lee et al., 2021; Loiseau et al., 2021) and mental strain (Dubey et al., 2020; Green, 2020; Orgilés et al., 2020; Ravens-Sieberer et al., 2021a), but may also negatively affect the so much needed support structures (youth organizations, youth welfare, professional counseling, and psychotherapy).

Not obvious but related to the points above, we asked children about their subjectively assessed risk posed by the coronavirus (SARS-CoV-2) and their source of information regarding the virus. On the one hand, this is interesting as it allows us to better understand the source of their fears and worries and on the other hand it relates to the legitimate question whether it was appropriate to vote for school closures for this youngest age group if scientific evidence indicates they are disproportionally less affected by SARS-CoV-2 infections themselves.

In our study, the risk of being hospitalized for a SARS-CoV-2 infection was estimated by children and adolescents at between 1 and 3%. At first, this does not sound high as compared to adult participants, who even estimated their personal risk for ICU admission after SARS-CoV-2 infection between at 11 and 18% (thereby overestimating it up to 97-fold) (Schabus et al., 2022). However, given current data (Langmeyer et al., 2020; Loiseau et al., 2021) which calculate the risk for hospitalization in this young age at or below 1 in 10,000 (<0.01%), this is still a 100-fold overestimation. Although it is acknowledged that such risk estimations are difficult to perform for human participants, it still draws a worrisome overall picture, namely, one of living in a subjectively life-threatening pandemic, and this for more than 24 months (as of March 2022).

With respect to the source of Corona-related information in our sample, we found that the youngest (6–10 year old) elementary school students primarily obtain their information from their parents and family (67.3%), whereas adolescents primarily responded to using “social media” or the internet as their main source of information (46.9%). This seems little surprising but reflects that the family is probably the strongest driver for subjective risk perception regarding COVID-19 in the youngest (as seen in our subgroup comparisons), whereas adolescents already seem to rely more on information they themselves look up in the internet; a strong and direct influence of public TV on risk perception as documented in the adults (Schabus et al., 2022) therefore plays a minor role in children and adolescents, although the risk perception of families themselves are likely strongly influenced by information provided by public TV and media resources.

Turning to the limitations of our study, we need to note that this was an *ad hoc* study with an online convenience sample given that signs accumulated that especially children and adolescents were widely overlooked in their needs, challenges, and subjective burden in Austria. Also, our own and earlier study on the psychosocial burden in the Austrian society only sampled adults aged 18 and above (Schabus et al., 2022); consequently, we decided to follow up with a tailored study for young children up to the age of 18 to address their specific situation and needs. The study is a snapshot between February and April 2021—that is, starting 2 weeks after the third lockdown ended—in Austria. Considering the results of Austrian data from other workgroups (Pieh et al., 2020, 2021; Dale et al., 2022), we fear a worsening of mental health (especially in girls) in the areas of well-being, depression, suicidal thoughts, and sleep, also after the end of our survey. In that same direction other groups report [Özlü-Erkilic et al. (2021), here for Turkey and Austria in 15–25 year olds] that, as the pandemic progressed, the fear for themselves and/or a family member to become infected and/or ruminations about COVID-19 were steadily increasing from wave to wave. Therefore, rather than demonstrating increasing resilience and coping skills over time, young people seem to be experiencing deteriorating mental health as the pandemic continues. In consequence, and despite the fact that our study was a “snapshot” in the midst of the pandemic, we believe that our data does not exaggerate the degree of burden in those vulnerable age groups between 6 and 18 years of age.

A strength of the study is certainly that the survey was advertised widely in public media and national television and therefore was sampled from a big cohort (*N* = 5,008) and even among the youngest, elementary school age children *n* = 949 valid answers were obtained. The quantitative data presented is complemented by plenty of qualitative data in the form of online written reports by children and adolescents. We there asked about home-schooling, annoyances during the pandemic, things they missed, and other unmentioned things that they want to speak out about the pandemic; we refer the interested reader to those (anonymized) data, which are available online (https://osf.io/sjt9g/). The data underline impressively how school children of all ages and adolescents thought and felt about the pandemic and how it affected their personal lives.

Altogether, and from a psychological point of view, it is obvious that we need to learn from mistakes in the past and act today to not later face a “lost generation” that was left alone with the challenges and burden of a worldwide pandemic and with the spotlight turned away from them for too long.

## Data availability statement

The data that support the findings of this study, the children’s unfiltered comments from the open questions of the questionnaire, as well as the questionnaire itself are openly available in “Open Science Framework” at: https://www.doi.org/10.17605/OSF.IO/SJT9G.

## Ethics statement

The Corona-related surveys were approved by the Ethics Committee of the University of Salzburg (EK-GZ 122013) and conducted in accordance with the Declaration of Helsinki with healthy volunteers. Written informed consent from the participants or their legal guardian/next of kin was not required to participate in this study in accordance with the national legislation and the institutional requirements.

## Author contributions

MS initiated the study and critically revised the manuscript and gave final approval for the version to be published. MS and E-SE were responsible for the conception of the manuscript and data collection. MS, E-SE, and SW carried out the file analysis and evaluation and wrote the manuscript. All authors contributed to the article and approved the submitted version.

## Footnotes

1 Bothe, K., Schabus, M., Eigl, E.-S., Kerbl, R., and Hoedlmoser, K. (in revision). *Self-reported changes in sleep patterns and behavior in children and adolescents during COVID-19*. Department of Psychology, University of Salzburg, Austria.
